# Influence of processing on flavonoid retention, transformation, and bioavailability in Tartary buckwheat (*Fagopyrum tataricum*): a review

**DOI:** 10.1016/j.fochx.2026.103872

**Published:** 2026-04-19

**Authors:** Hong-Ju He, Guanglei Li, Xingqi Ou, Amer Ali Mahdi, Mohammed Obadi

**Affiliations:** a School of Food Science, Henan Institute of Science and Technology, Xinxiang 453003, China; b School of Life Science & Technology, Henan Institute of Science and Technology, Xinxiang 453003, China; c Department of Food Science and Nutrition, Faculty of Agriculture, Food, and Environment, Sana'a University, Sana'a, Yemen

**Keywords:** Tartary buckwheat, Flavonoids, Milling, Germination, Fermentation, Thermal processing

## Abstract

Buckwheat is a traditional food and medicinal crop valued for its nutritional composition and high levels of bioactive compounds. Among buckwheat species, Tartary buckwheat is particularly rich in flavonoids, especially rutin and quercetin, which are associated with antioxidant, hypoglycemic, lipid-lowering, antihypertensive, and anti-inflammatory effects. However, the concentration and stability of these compounds are strongly influenced by processing. This review summarizes how non-thermal processes (milling, soaking, germination, and fermentation) and thermal treatments (boiling, steaming, roasting, and extrusion) affect the distribution, transformation, and retention of flavonoids in Tartary buckwheat. The evidence shows that optimized processing can enhance flavonoid bioavailability, reduce antinutritional factors, and improve product quality, enabling the development of functional Tartary buckwheat foods with improved nutritional and health-promoting value. The review concludes that standardised processing conditions and better understanding of flavonoid transformations are essential to maximize health benefits and support industrial application of Tartary buckwheat products.

## Highlights

• Tartary buckwheat is a major dietary source of rutin and health-promoting flavonoids.
• Impact of processing on the bioactive components of Tartary buckwheat was reviewed.
• The proper processing can improve the nutritional value of Tartary buckwheat.
• Problems and challenges to apply these technologies are presented.

## Graphical abstract

**Figure:**
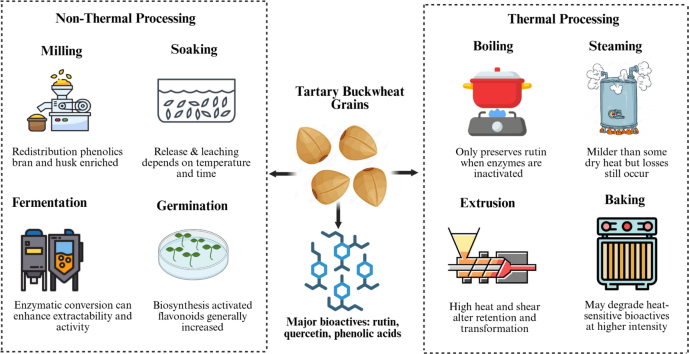

## 1 Introduction

Buckwheat is a pseudocereal widely valued in human nutrition due to its high content of phenolic compounds and associated physiological benefits. Among species of the *Fagopyrum* genus, only Tartary buckwheat (*Fagopyrum tataricum* Gaertn) and common buckwheat (*Fagopyrum esculentum* Moench) are widely consumed as food crops (Jha et al., 2024). Buckwheat cultivation plays an essential role in high-altitude and mountainous regions where other staple crops are difficult to grow, with major production concentrated in countries such as China, Russia, Japan, Poland, France, Canada, and the United States. China remains the leading producer, contributing a significant share of global cultivation area and output (Q. Wang et al., 2024). Tartary buckwheat has attracted increasing attention because of its superior nutritional and functional properties. It is rich in flavonoids, proteins, dietary fiber, vitamins, and minerals, making it a promising functional food ingredient (Ruan et al., 2022; Zou et al., 2023). Among its bioactive compounds, rutin and quercetin are predominant and largely responsible for its antioxidant capacity, which is typically higher than that of common buckwheat (Tien et al., 2018). In addition, buckwheat-based ingredients have been reported to contribute to the mitigation of acrylamide-related hazards in baked products, as bioactive compounds such as rutin and quercetin may help counteract acrylamide-induced DNA damage and related toxic effects (Vogrinčič & Žegura, 2025). Beyond antioxidant effects, studies have reported anti-inflammatory, hypoglycemic, lipid-lowering, and hepatoprotective activities associated with Tartary buckwheat consumption (Zhong et al., 2022; Zou et al., 2023). These effects are partly linked to interactions between flavonoids and other food matrix components, including starch, which influence carbohydrate digestibility and metabolic responses (T. Zhou et al., 2025; Y. Zhou et al., 2023; Y. Zhou et al., 2021). In addition to its nutritional benefits, Tartary buckwheat contains certain antinutritional factors, and its composition can vary with cultivar selection and cultivation environments (Kreft et al., 2022; T. Zhou et al., 2025). Processing technologies strongly influence the nutritional and functional properties of Tartary buckwheat. Techniques such as milling, soaking, germination, fermentation, boiling, steaming, roasting, and extrusion modify flavonoid composition, antioxidant capacity, and sensory characteristics of buckwheat products. For example, fermentation and related treatments can enhance bioactive compound availability by releasing bound phenolics (R. Zhang et al., 2024). Although several reviews have summarized buckwheat bioactive compounds (Huda et al., 2021; Podolska et al., 2021; Zou et al., 2023), a comprehensive evaluation of how different processing methods influence flavonoid composition and transformation remains limited. Therefore, this review focuses on the effects of both non-thermal processes, including soaking, milling, germination, and fermentation, and thermal treatments such as boiling, steaming, roasting, and extrusion on flavonoid composition and related functional properties of Tartary buckwheat. The aim is to support improved processing strategies and the development of health-oriented buckwheat-based products.

## 2 Bioactive compounds

Tartary buckwheat is a plant rich in diverse bioactive compounds. A wide range of chemical constituents have been identified, which can be broadly categorized based on their chemical structures. These primarily include flavonoids, phenolic acids and their derivatives, tannins, fagopyrins, triterpenoids, steroids, and stilbenes (Huda et al., 2021). The family, basic skeleton, compound names, and analytical techniques used for their characterization are summarized in Table 1. The following is an overview of the major compounds identified in Tartary buckwheat.

**Table 1:** Overview of bioactive constituents identified in various Tartary buckwheat seeds and their derived products, along with the analytical techniques used for their characterization.

Family	Basic skeleton	Compounds	Identification methodology	References
Flavonols		3-O-rutinoside-3′-O-β-glucopyranoside, rutin, kaempferol 3-rutinoside	ESI-MS/MS, HSCCC, NMR	(Borovaya et al., 2020; Jiang et al., 2015; J. M. Lee et al., 2013)
Flavones		Vitexin, orientin, isovitexin, isoorientin	HPLC-PDA, NMR	(J. M. Lee et al., 2013; Mansur et al., 2020)
Flavan-3-ols		Catechin, epicatechin, epicatechin gallate, epigallocatechin	UPLC-MS-MS, RP-HPLC, HPLCFTICR-MS	(Aleksenko et al., 2022; Borovaya et al., 2020)
Phenolic acids and their derivatives Hydroxybenzoic acids		*p*-hydroxybenzoic acid, *p*-coumaric acid, gallic acid, caffeic acid, vanillic acid, syringic acid, chlorogenic acid, 4-hydroxy-3-methoxy benzoic acid	HPLC-DAD-ESIMS, NMR	(X.-D. Guo et al., 2011; L.-X. Peng et al., 2017; Uddin et al., 2013)
Phenylpropanoid glycosides		1,3,6,60-tetra-feruloyl sucrose, 1,3,6-tri-p-coumaroyl-60- feruloyl sucrose 3,6-di-pcoumaroyl- 1,60-di-feruloyl sucrose, 1,3,60-tri-feruloyl-6-p-coumaroyl sucrose, tatarisides A-G, diboside A	HPLC-PDA/LIT-FTICR-MS, NMR	(Q. Ren et al., 2013; C. Zheng et al., 2012; Zhu, 2016)
Steroids		β-sitosterol, β-sitosterol-3-O-glucoside, 6-hydroxystigmasta	HPLC-EI-MS, NMR	(J. M. Lee et al., 2013; Ruan et al., 2022)
Triterpenoids		oleanolic acid	HPLC-ESI-MS, NMR, GC–MS	(Bilal Pirzadah et al., 2016; J. M. Lee et al., 2013)

Note: high-speed counter-current chromatography (HSCCC), electrospray ionization tandem mass spectrometry (ESI-MS/MS); High-Performance Liquid Chromatography (HPLC), ultra-performance liquid chromatography (UPLC), photodiode array (PDA), mass spectrometry (MS),

### 2.1 Flavonoid

Flavonoids are a major group of bioactive compounds abundantly present in buckwheat seeds, especially in Tartary buckwheat. These compounds contribute significantly to the nutritional and medicinal value of buckwheat, providing antioxidant, anti-inflammatory, anticancer, anti-diabetic, and cardiovascular protective effects (Choi et al., 2021). Tartary buckwheat seeds are particularly rich in flavonoids, with rutin, quercetin, nicotiflorin, and kaempferol identified as the main components, together accounting for 73.05 to 81.79% of the total flavonoid content (TFC) (Ke et al., 2023). Among these, rutin and quercetin are especially notable for their health benefits. Their contents vary among different cultivars, with rutin ranging from 4354.1 to 17,196.01 μg g − 1 DW and quercetin from 8.9 to 611.7 μg g − 1 DW (Choi et al., 2021). Compared to common buckwheat, Tartary buckwheat seeds contain much higher levels of rutin, reaching approximately 0.8 to 1.7% dry weight, while common buckwheat contains only about 0.01% (Borovaya & Klykov, 2020; Ke et al., 2023). The TFC varies depending on species, variety, and environmental conditions. Tartary buckwheat flour often shows higher concentrations than common buckwheat, with some cultivars reaching up to 22.74 mg g^−1^ of total flavonoids and 2.38 mg g^−1^ of quercetin (Qin et al., 2010). The highest concentrations are typically found in the bran and outer seed layers. Among the major flavonols found in Tartary buckwheat are the aglycones quercetin and kaempferol, which are usually present in glycosylated forms such as quercetin-3-O-rutinoside (rutin) and kaempferol-3-O-rutinoside (*J*. M. Lee, Lee, et al., 2013; X. L. Li & Li, 2012; L.-X. Peng et al., 2013). Rutin is considered a key bioactive compound due to its wide range of beneficial effects (M. Liu et al., 2018; L.-X. Peng et al., 2013). A comprehensive metabolite profiling study identified 234 flavonoids in Tartary buckwheat seeds, including 10 isoflavones, with 80 compounds showing significant changes during seed development (H. Li et al., 2019). Another study using liquid chromatography-mass spectrometry detected 58 flavonoid compounds, including 42 flavonols, 10 flavanols, three flavanones, one isoflavone, one anthocyanidin, and one proanthocyanidin (Peng Wang et al., 2023). Specific flavonols such as kaempferol-3-O-hexoside, kaempferol-7-O-glucoside, and naringenin-O-hexoside were found to strongly correlate with TFC and antioxidant capacity. Additionally, quercetin-3-O-rutinoside-3’-O-β-glucopyranoside and the compound umbelliferone have been isolated from Tartary buckwheat seeds (Li & Li, 2012; Zheng Feng et al., 2011). The chemical structures of flavonoids and their derivatives isolated from Tartary buckwheat seeds are determined through various spectroscopic analyses. Techniques such as nuclear magnetic resonance (NMR), including both ^1^H NMR and ^13^C NMR, are crucial for elucidating the intricate atomic arrangements within these molecules (Lee, Lee*,* et al., *2013*; Shyamal et al., 2020). Mass spectrometry (MS), particularly electrospray ionization tandem mass spectrometry (ESI-MS/MS), is widely employed to ascertain the molecular weight and fragmentation patterns, which are vital for accurate structural identification. For example, the structures of kaempferol, quercetin, kaempferol-3-O-rutinoside, and quercetin-3-O-rutinoside were elucidated based on ^1^H NMR, ^13^C NMR, and MS data (Lee, Shen*,* et al., *2013*). In one study, ESI-MS/MS was specifically used to obtain ion fragments and mass-to-charge ratios for the detailed identification of four flavonoid compounds: quercetin 3-O-rutinoside-3’-O-β-glucopyranoside, rutin, kaempferol 3-O-rutinoside, and quercetin (Li & Li, 2012). Other spectroscopic methods like ultraviolet (UV) and infrared (IR) spectroscopy also contribute to confirming the structures of isolated compounds (Zheng Feng et al., 2011). These analytical approaches confirm that flavonols from Tartary buckwheat typically possess a core flavonol structure with various sugar moieties attached, commonly at the 3-O position (*J*. M. Lee et al., 2013). Flavonoid accumulation is developmentally regulated and changes during seed maturation (Ke et al., 2023; Song et al., 2016). Besides their roles in human health, flavonoids play important functions in plant growth, reproduction, and defense mechanisms (Borovaya & Klykov, 2020).

### 2.2 Phenolic acids

Tartary buckwheat seeds are rich in a variety of phenolic acids, contributing to their notable health benefits. The most prominent phenolic acids identified are p-hydroxybenzoic acid, ferulic acid, and protocatechuic acid (X.-D. Guo et al., 2011; Wahlsten, 2019). These three acids are particularly abundant, collectively accounting for approximately 83–88% of the total phenolic acid content in Tartary buckwheat (Wahlsten, 2019). Specifically, *p*-hydroxybenzoic acid is a major phenolic acid, comprising 65.54% of the phenolic acids found in Tartary buckwheat hulls (Ruan et al., 2022). Beyond these primary compounds, other important phenolic acids detected in Tartary buckwheat seeds include *p*-coumaric acid, gallic acid, caffeic acid, vanillic acid, and syringic acid (X.-D. Guo et al., 2011). Chlorogenic acid is also present in Tartary buckwheat, with significant amounts measured in the flower of certain cultivars (Uddin et al., 2013). Studies have identified a total of six phenolic compounds in Tartary buckwheat flour and bran (L.-X. Peng et al., 2017). Additionally, other phenolic compounds such as 4-hydroxy-3-methoxy benzoic acid and epicatechin have been identified in various parts of the plant, including flowers, leaves, stems, and roots (Uddin et al., 2013). X.-D. Guo et al. (2012) analyzed the phenolic acid content in four fractions of Tartary buckwheat: hulls, coarse bran, fine bran, and core flour. The study showed that the levels of free phenolic acids were significantly higher than those of bound forms across all samples. Eight phenolic acids were detected in each part: p-hydroxybenzoic acid, protocatechuic acid, chlorogenic acid, gallic acid, ferulic acid, *p*-coumaric acid, syringic acid, and vanillic acid. Protocatechuic acid accounted for 65.54% of total phenolic acids in the hulls, making it the dominant compound in that fraction, while *p*-hydroxybenzoic acid was the major phenolic acid in bran and core flour, contributing 58.61% and 78.82% respectively. During the germination process, the content of phenolic acids such as gallic acid and chlorogenic acid increases, indicating that germination can enhance the phenolic profile of Tartary buckwheat (Y. Chen et al., 2022). Furthermore, the phenolic content in Tartary buckwheat is influenced by the specific part of the plant, with flowers containing higher levels of phenolic compounds compared to leaves, stems, and roots (Uddin et al., 2013). The concentration of these phenolic acids varies significantly among different cultivars and geographical origins, reflecting the diversity in phenolic composition across Tartary buckwheat varieties (T. Liu et al., 2024; Uddin et al., 2013).

### 2.3 Quinones

Quinones are a class of compounds featuring a quinonoid structure and are commonly found in various medicinal plants. Within Tartary buckwheat seeds, the primary quinones identified as bioactive compounds include fagopyrins and emodin (Luthar et al., 2020). These compounds are part of the broader category of (poly)phenolics found abundantly in both common and Tartary buckwheat. Fagopyrins are specifically noted for being light-sensitive naphthodianthrones, sharing structural similarities with hypericin, and possessing photoreactive properties (Kim et al., 2021; Luthar et al., 2020). Emodin, on the other hand, is an anthraquinone and has been recognized for its potential antiviral effects. The presence of these quinones contributes to the overall biological activity of Tartary buckwheat, supporting its use for various health-promoting purposes (Zou et al., 2023). Current research on quinones in Tartary buckwheat mainly focuses on their extraction, identification, and evaluation of biological activities. Xingqiang Wu et al. (2015) employed selective accelerated solvent extraction (with methanol: acetone = 50:50, *v*/v as the solvent and silica gel as the adsorbent), combined with ultra-performance liquid chromatography with diode array detection (UPLC-DAD), to simultaneously quantify six anthraquinones: chrysophanol, aloe-emodin, rhein, emodin, physcion, and emodin methyl ether. These anthraquinones are likely present in the seeds in glycosylated forms. L.-X. Peng et al. (2013) carried out a simple identification of emodin in Tartary buckwheat seeds using HPLC-DAD. The content of emodin in four Chinese Tartary buckwheat cultivars ranged from 1.72 to 2.71 mg kg^−1^.

### 2.4 Phenylpropanoid glycosides

Phenylpropanoid glycosides are among the various bioactive compounds found in Tartary buckwheat (Zou et al., 2023). While specific individual phenylpropanoid glycosides in the grains are not extensively detailed in the provided documents, the broader category of phenylpropanoid glycosides is acknowledged as a significant group of secondary metabolites in Tartary buckwheat (X. Li et al., 2017; Zou et al., 2023). A diverse range of phenylpropanoid glycosides has been identified in the roots and seeds of Tartary buckwheat, with twelve distinct compounds reported. In the seeds of Tartary buckwheat, four specific phenylpropanoid glycosides have been detected: 1,3,6,6′-tetra-feruloyl sucrose, 1,3,6-tri-*p*-coumaroyl-6′-feruloyl sucrose, 3,6-di-*p*-coumaroyl-1,6′-di-feruloyl sucrose, and 1,3,6′-tri-feruloyl-6-*p*-coumaroyl sucrose. These compounds were identified using high-performance liquid chromatography coupled with photodiode array detection and Fourier transform ion cyclotron resonance mass spectrometry (Huda et al., 2021; Zhu, 2016). In addition, several phenylpropanoid glycosides unique to Tartary buckwheat roots have been identified, including seven tatarisides (tatariside A to tatariside G) and diboside A (C. Zheng et al., 2012).

### 2.5 Steroids

Steroids constitute a broad category of cyclopentane perhydrophenanthrene derivatives that are widely distributed in the biological world, including the plant kingdom (Zou et al., 2023). In Tartary buckwheat seeds, a diverse array of these compounds has been identified, contributing to the plant's overall bioactive profile (Zou et al., 2023). Specifically, five distinct steroids have been determined in the seeds (C.-C. Lee, Shen, et al., 2013; Lv et al., 2017). These include important phytosterols and triterpenoids (Ruan et al., 2022). Notable phytosterols identified are β-sitosterol and its glucoside derivative, β-sitosterol-3-O-glucoside (C.-C. Lee et al., 2013). β-sitosterol, a common plant steroid, is found in Tartary buckwheat and is recognized for its presence in the seeds. Other steroid derivatives like 6-hydroxystigmasta compounds are also present, further diversifying the steroidal composition (Ruan et al., 2022). The presence of these steroids, particularly phytosterols, is linked to the potential health benefits associated with Tartary buckwheat consumption. Quantitative analyses using techniques such as High-Performance Liquid Chromatography (HPLC) have established that the β-sitosterol content across 34 different Tartary buckwheat cultivars ranges from approximately 4.1 to 65.3 mg/100 g. When comparing different parts of the seed, the bran fraction consistently shows the highest concentration of β-sitosterol, reaching up to 116 mg/100 g. Conversely, the lowest concentration is found in the center flour (L. Peng et al., 2012).

### 2.6 Triterpenoids

Triterpenoids are another class of bioactive compounds identified in Tartary buckwheat grains (Zou et al., 2023). A significant finding from the seeds of *Fagopyrum tataricum* is the isolation of oleanolic acid for the first time (J. M. Lee et al., 2013). This compound is recognized as a triterpenoid alongside others like β-sitosterol and β-sitosterol-3-O-glucoside, which, although often classified as phytosterols (steroids), are sometimes broadly discussed in the context of triterpenoid-related compounds due to structural similarities and biosynthetic pathways (J. M. Lee et al., 2013). The major compounds identified in Tartary buckwheat extracts, including groat extract, were found to include terpenoids, along with fatty acids, hydrocarbons, steroids, esters, organic acids, and aldehydes, all possessing excellent pharmaceutical properties (Bilal Pirzadah et al., 2016). The comprehensive presence of triterpenoids, alongside other bioactive compounds, further underscores the significant medicinal and nutritional value of Tartary buckwheat grains (Zou et al., 2023).

## 3 Effects of processing methods on Tartary buckwheat flavonoids

Whole grain Tartary buckwheat typically requires processing prior to consumption, primarily to enhance the taste and flavor of the grain and its derivatives. Processing also helps reduce moisture content, eliminate harmful microorganisms, and deactivate biological enzymes, thereby improving product stability, safety, and extending shelf life. Furthermore, processing can alter the tissue structure of Tartary buckwheat and deactivate certain antinutritional factors (such as protease inhibitors and rutin-degrading enzymes), thereby increasing the bioavailability of essential nutrients. Common processing techniques for Tartary buckwheat include non-thermal methods such as milling, soaking, germination, and fermentation, as well as thermal methods like boiling, steaming, roasting, and extrusion. The effect of these processing methods on flavonoids like rutin and quercetin depends on both the processing technique and the specific parameters used. Table 2 provides a summary of the impact of common processing methods on the active components of Tartary buckwheat.

**Table 2:** Studies Investigating the Impact of Processing Methods on the Flavonoid Profiles in Tartary Buckwheat.

Processing type	Processing conditions	Analysis/analyte	Changes in flavonoid compounds	Reference
Milling	Quadrumate Senior Mill, the fractions were classified into seven fine flours, three coarse flours, four small semolina, two large semolina, six bran fractions, and pure husks.	TPC, TFC, and DPPH	(↑) TPC (155.23–1024.19 mg GAE/100 g); (↑) TFC (122.83–734.89 mg QEQ/100 g); (↑) DPPH (11.67–25.93%)	(Mazahir et al., 2022)
	Shear crushing, airflow comminution, and wet grinding	Rutin, quercetin, TFC, TPC, DPPH, ABTS and ·OH	(↓) Rutin (3.90 → 0–3.16 g/100 g); (↑) quercetin (1.03 → 0.97–2.18 g/100 g); (↓) TFC (9.50 → 8.69–9.50 g/100 g); (∼) DPPH (63.73 → 60.74–64.07%); (↑) ABTS (89.73 → 90.16–92.98%); (↑)·OH scavenging (26.75 → 34.54–86.62%)	(Q. Xu et al., 2021)
	Ultrafine milling, roller milling, stone milling, and wet milling.	Quercetin, rutin, TPC, TFC, DPPH and TRP	(↑) TPC (611.3–1682.2 mg GAE/100 g DW); (↑) TFC (416.7–722.7 mg rutin eq./100 g DW); (↑) quercetin (3.11–553.7 mg GAE/100 g DW);(↑) DPPH (10.3–14.9 mmol Trolox eq./100 g DW); (↑) TRP (471.0–741.3 mg VC eq. per 100 g DW)	(F. Liu et al., 2018)
Soaking	Soaking (12–24 h at room temperature)	TPC and antioxidant activity	(↑) TPC (210.31 → 251.87–280.81 mg GAE/100 g); (↑) antioxidant activity (31.69 → 52.53–55.46%)	(Thakur et al., 2021)
	Soaking (1 h/19 °C)	Polyphenol compounds	(↑) Catechin (20.87–34.89 mg kg^−1^ DW); (↓) Epicatechin (56.51–36.85 mg kg^−1^ DW); (∼) Epicatechin gallate (9.80–9.33 mg kg^−1^ DW); (↑) Rutin (52.48–55.78 mg kg^−1^ DW)	(Kalinová et al., 2019)
	Soaking at temperatures ranging from 51 °C to 100 °C for 20 min	Flavonoid compounds	(↓) rutin (30.602–28.209 mg g^−1^), (↓) quercetin (0.236–0.042 mg g^−1^)	(Brajčič et al., 2020)
Germination	Germination: 25 °C for 0, 0.5, 1, 1.5, 2, 2.5, 3, 4, 5, 6, 7, 8, 9 and 10 days	Polyphenol compounds, antioxidant activity	At 10 days of buckwheat germination (↑) Rutin (1585–2453 mg/100 g DW), (↓) gallic acid (2652–188.26 mg/100 g DW), (↑) chlorogenic acid (7.110–96.252 mg/100 g DW), (↑) 2,3,4-trihydroxybenzoic acid (11.278–46.502 mg/100 g DW), (↓) quercetin (43.790–33.313 mg/100 g DW)	(Y. Chen et al., 2022)
	Germination: 25 °C and 75% humidity for 0, 1, 3, 5, 7, and 9 days	TPC, TFC, quercetin, and rutin	At 9-day germination period (↑) TPC (324.7–553.4 mg g^−1^ DW), (↑) TFC (72.5–108.2 mg g^−1^ DW), (↑) rutin (36.37–42.24 mg g^−1^ DW)	(S.-C. Ren & Sun, 2014)
	Germination: 30 °C and 85% humidity for 1, 2, 3, 4, 5, 6, and 7 days	Phenols, antioxidant compounds and antioxidant capacities	At 7-day germination period (↑) DPPH (2.6–5.4 μmol TE/g), (↑) ABTS (0.9–2.2 μmol TE/g), (↑) superoxide free radicals (5.8–10.9 μmol TE/g) (↑) carotenes (3.6–10.7 mg CE/g), (↑) TFC (3.72–19.53 mg rutin/g), (↑) rutin (2.78–11.34 mg g^−1^), and (↑) quercetin (0.49–3.98 mg g^−1^)	(X. Zhou et al., 2015)
	Germination: 25 °C and 90% humidity for 12, 24, 36, 48, 60, and 72 h	TPC, TFC, DPPH, TEAC, FRAP, and ORAC	At 72-day germination period (↑) TPC (3.03–8.42 mg GAE/g), (↑) TFC (4.17–11.69 mg RE/g), (↑) DPPH (6.32–24.50 mmol TE/kg), (↑) TEAC (13.01–46.01 mmol TE/kg), (↑) FRAP (18.07–63.94 mmol Fe^2+^ /kg), and (↑) ORAC (80.50–256.67 mmol TE/kg)	(G. Zhang et al., 2015)
Fermentation	Fermented Tartary buckwheat grain with *Ganoderma lucidum* at 26 °C for 21 days	TPC and TFC	(↑) TPC (261.19–558.17 mg GAE/100 g DW) and (↑) TFC (534.97–1624.89 mg QE/100 g DW)	(R. Zhang et al., 2024)
	Germinated Tartary buckwheat grain for 2 days at 18 °C and then fermented with *Bacillus* spp., *Lactobacillus* spp. and *Bifidobacter Lum* spp.; at 30 °C, 37 °C, and40 °C for 48–72 h	TPC, TFC, and antioxidant activity	(↑) Rutin (0.30–0.97 g kg^−1^), (↑) quercetin (0.49–2.53 g kg^−1^), (↑) TPC (2.2–3.41% DW), (↑) TFC (0.14–4.28% DW), (↑) DPPH (7.75–45.56% DW)	(Ratha et al., 2017)
	Cooked Tartary buckwheat grain for 10 min acidified to pH 4.5–5.0 with lactic acid and fermented with *Rhizopus oligosporus* at 35 °C for 40 h	Phenols and antioxidant activity	(↑) DPPH (9.46–10.79 μmol trolox/g DM), (↑) ABTS (27.20–42.65 μmol trolox/g DM), (↑) soluble phenols (6.18–7.47 mg g^−1^ DM)	(Starzyńska-Janiszewska et al., 2016)
	Fermentation (Monascus anka) at 28 °C for 8 days	Polyphenol compounds	(∼) Gallic acid (85.9–85.8 μg/g DW), (↓) p-hydroxybenzoic acid (44.9–431.8 μg/g DW), (↓) 5- cafeoylquinic acid (55.5–53.5 μg/g DW), (↑) rutin (15,037.3–23,278.8 μg/g DW), (↑) kaempferol-3-Orutinoside (1249.7–2226.5 μg/g DW), (↑) apigenin (1.1–25.6 μg/g DW), (↑) syringic acid (43.9–50.1 μg/g DW), (↑) ferulic acid (35.7–87.2 μg/g DW), (↑) quercetin (327.0–450.4 μg/g DW), (↑) kaempferol (13.5–18.8 μg/g DW)	(S. Chen et al., 2021)
Boiling	Boiling by domestic cooker at 100 °C for 10 min	TPC, TFC, FRAP and ABTS	(↑) TPC (6.72–10.42 mg GAE/g DW), (↑) TFC (3.81–5.89 mg CAE/g DW), (↑) FRAP (5.87–10.46 mmol Fe^2+^E/100 g DW), (↑) DPPH (16.84–17.56 TE/g DW), and (↑) ABTS (43.25–52.12 μmol TE/g DW), (↓) quercetin (128.56–115.50 mg kg^−1^), (↓) rutin (7623.26–6975.48 mg kg^−1^), (↓) quercitrin (23.63–18.67 mg kg^−1^)	(Y. Liu et al., 2019)
	Boiling by domestic cooker at 100 °C for 5 min	TPC, TFC, DPPH, and ABTS	(↓) TPC (27.73–18.27 mg g^−1^), (↓) TFC (95.76–61.00 mg g^−1^), (↓) DPPH (69.19–49.19%), (↓) ABTS (56.48–37.22%)	(N. Wang et al., 2024)
	Boiling by domestic cooker at 100 °C for 30 min	TFC	(↓) TFC (2.49–1.54 g/100 g DM)	(Deng et al., 2015)
	Boiling by domestic cooker at 100 °C for 5, 10, and 15 min	TPC, TFC, DPPH, and ABTS	(↑) TPC (1.40–2.30 g TAE/100 g), (↑) TFC (0.42–1.38 g CE/100 g), (↑) DPPH (1.6–3.1 AAE/100 g), and (↑) ABTS (7.1–12.5 AAE/100 g)	(Jin et al., 2020)
	Boiling by water bath at 100 °C for 15 min	Quercetin, and rutin	(↑) quercetin (0.61–1.83 mg g^−1^ DW), (↓) rutin (25.65–22.27 mg g^−1^ DW)	(L. Wang et al., 2025)
	Soaking in boiling water 100 °C for 5–20 min	TPC, TFC, and FRAP	(↓) TPC (0.84 → 0.58–0.69 mg GAE g^−1^); (↓) TFC (0.96 → 0.56–0.68 mg QE g^−1^); (↓) FRAP (70.21 → 26.44–41.19 mM g^−1^)	(Roy et al., 2019)
Steaming	Pre-soaked+Steaming (100 °C /10 min)	Flavonoid compounds	(↓) quercetin (128.56–53.92 mg kg^−1^), (↓) rutin (7623.26–6191.02 mg kg^−1^), (↓) quercitrin (23.63–11.73 mg kg^−1^)	(Y. Liu et al., 2019)
	Steaming at 120 °C for 20 min	Polyphenol compounds	(↓) Gallic acid (85.9–72.9 μg/g DW), (↑) 4-hydroxybenzoic acid (44.9–58.5 μg/g DW), (↑) 5-cafeoylquinic acid (55.5–57.5 μg/g DW), (∼) rutin (15,037.3–15,046.9 μg/g DW), (↑) kaempferol-3-O-rutinoside (1249.7–1264.8 μg/g DW), (↓) apigenin (1.1–0.9 μg/g DW), (↑) syringic acid (43.9–44.5 μg/g DW), (↓) ferulic acid (35.7–30.7 μg/g DW), (↑) quercetin (327.0–339.3 μg/g DW), (∼) kaempferol (13.5–13.4 μg/g DW), (↓) ABTS (96.6–59.8 μmol TE/g DW), (↓) DPPH (86.4–82.0 μmol TE/g DW), (↓) FRAP (56.5–47.5 mM FeS(II) E/g DW)	(S. Chen et al., 2021)
	Steam explosion (1.5 MPa/ 60 s)	Flavonoid compounds and antioxidant activities	(↑) TPC (23.98–28.32 mg GAE/g DW), (↑) TFC (10.86–13.18 mg RE/g DW), (∼) ORAC (1122.54–1120.33 μmol TE/g DW)	(W. Li et al., 2020)
	Steaming in combination with oven-drying. Steaming: 100 °C for 10 min and then dried in the oven at 80 °C	TFC and antioxidant activity	(↑) TPC (31–34 mg g^−1^ DW), (↑) rutin (18–21 mg g^−1^ DW), and (↓) quercetin (0.55–0.14 mg g^−1^ DW)	(Tang et al., 2024)
Roasting	Roasting at 180 °C for 15 min	TFC and antioxidant activity	(↑) TPC (31–26 mg g^−1^ DW), (↑) rutin (18–12 mg g^−1^ DW), and (↓) quercetin (0.55–0.30 mg g^−1^ DW)	(Tang et al., 2024)
	Roasting at 175, 200, 225, and 250 °C for 5 and 10 min	Phenolic compounds and antioxidant activities	At roasting (175 °C, 10 min): (↑) TPC (2.05–5.94 mg TAE g^−1^), at roasting (225–250 °C, 10 min): (∼) TPC (2.05–2.77 mg TAE g^−1^)	(M.-H. Lee et al., 2014)
	Pre-soaked + Roasting (120 °C for 30 min)	TPC, phenolic compounds and antioxidant activities	(↓) Gallic acid (85.9–53.7 μg/g DW), (↑) 4-hydroxybenzoic acid (44.9–90.2 μg/g DW), (↑) 5-cafeoylquinic acid (55.5–67.1 μg/g DW), (↓) rutin (15,037.3–11,614.8 μg/g DW), (↓) kaempferol-3-O-rutinoside (1249.7–937.3 μg/g DW), (↓) apigenin (1.1–0.1 μg/g DW), (↓) syringic acid (43.9–32.4 μg/g DW), (↑) ferulic acid (35.7–38.7 μg/g DW), (↓) quercetin (327.0–256.5 μg/g DW), (↓) kaempferol (13.5–9.7 μg/g DW), (↓) ABTS (96.6–57.8 μmol TE/g DW), (↓) DPPH (86.4–82.5 μmol TE/g DW), (↓) FRAP (56.5–53.3 mM FeS(II) E/g DW)	(S. Chen et al., 2021)
	Infrared roasting (130–170 °C for 10 min)	TPC, TFC, TAA, rutin, quercetin	(↓) TPC (8.90–14.72 → 5.46–12.32 mg GAE g^−1^ DW); (↓) TFC (23.74–28.67 → 17.62–23.31 mg RE g^−1^ DW); (↓) TAA (11.37–12.74 → 6.84–11.53 μmol TE g^−1^ DW); (↓) rutin (16.91–26.60 → 15.11–25.26 mg g^−1^ DW); (↓) quercetin (1.19–2.20 → 0.42–2.11 mg g^−1^ DW)	(Bhinder et al., 2019)
	Roasting (200 °C / 50 s)	TPC, TFC, antioxidant capacity	(↓) TPC (12–16 → 8–12 mg GAE g^−1^ DW); (↓) TFC (21–26 → 19–24 mg RE g^−1^ DW); (↓) antioxidant capacity (60–85 → 45–70 FRAP units)	(Ma et al., 2020)
Extrusion	Temperature: 40–160 °C; Feeding rate: 10–40 g/min; Feed moisture: 30–40%	TFC, rutin, quercetin	(↓) TFC (19.93 → 11.81–17.61 mg g^−1^ DW), (↓) rutin (18.40 → ND–15.42 mg g^−1^ DW), (↑) quercetin (0.07 → 0.18–8.21 mg g^−1^ DW)	(Z. Zhang et al., 2022)
	Temperature: 120–160 °C; screw speed: 120–180 rpm	Rutin	(↑) rutin (1.20–10.32 mg g^−1^ DW)	(D. Li et al., 2008)
	Extrusion temperature: 95–115 °C; pressure: 15 bar; screw speed: 160 rpm; ejection time after extrusion: 60–90 s; yeast addition: 0–2%	TPC, TF, rutin and DPPH	(↑) TPC (35–53 → 50–95 mg/100 g); (↑) TFC (64–91 → 83–181 mg/100 g); (↑) rutin (1.65–3.96 → 2.36–4.23 mg/100 g); (↑) DPPH (26–39 → 33–55%)	(Park et al., 2022)
	Individual extrusion of Tartary buckwheat (IE) vs mixed extrusion with adzuki bean (ME), temperature 120 °C, feed moisture content 14%, feeding rate 120 kg/h, and the screw speed 590 rpm	TPC,TFC, rutin and quercetin	(↑) TPC (2.67–5.09 mg g^−1^ DW); (↑) TFC (2.06–6.37 mg g^−1^ DW); (↑) rutin (1.48–5.69 mg g^−1^ DW); (↓) quercetin (0.09–0.04 mg g^−1^ DW)	(Z. Zhang et al., 2023)

Notes: (↑), means increased; (↓), means decreased; (∼), means no change. TAC, total anthocyanin content; TFC, total flavonoid content; TPC, total phenolic content; FRAP, ferric reducing antioxidant power; TF, total flavonoid; ORAC, oxygen radical absorbance capacity; TAA, total antioxidant activity; TRP, total reducing power.

### 3.1 Non-thermal processing

#### 3.1.1 Milling

Milling significantly modifies the distribution and concentration of phenolic compounds in Tartary buckwheat, with outer grain fractions such as bran and husk consistently showing higher concentrations than refined flours. The localization of rutin, quercetin, and other flavonoids in the outer layers explains why bran and husk fractions exhibit the highest total phenolic content (TPC), TFC, and antioxidant activity, while light flour shows the lowest values (Luthar et al., 2020; Sinkovič et al., 2021). Hung and Morita (2008) demonstrated through gradual milling of whole buckwheat grains into graded flour fractions that rutin increased from 2.5 μg/g in inner fractions to 389.9 μg/g in the outermost fractions, while ferulic acid rose from 2.5 μg/g to 609.5 μg/g, alongside significant increases in antioxidant capacity. Similarly, Hung et al. (2009) observed that phenolics and antioxidant activity progressively increased from the inner to the outer flour fractions obtained from grain milling, underscoring the nutritional value of bran-rich layers. X.-D. Guo et al. (2012) confirmed that fine bran fractions contained the highest free phenolic and flavonoid contents, with rutin levels reaching 7.43% of dry weight, while the hull fraction was rich in bound phenolics, with protocatechuic acid accounting for 65.54% of hull phenolics. In bran and light flour fractions, *p*-hydroxybenzoic acid was dominant (58.61–78.82%), and both rutin and p-hydroxybenzoic acid correlated strongly with antioxidant activity (*r* ≥ 0.98, *p* < 0.01). Mazahir et al. (2022) further demonstrated that Tartary buckwheat husk, bran flour, coarse flour, and fine flour milling fractions differed significantly, with husk containing the highest TPC (2101.42 mg GAE/100 g) and TFC (1233.99 mg QE/100 g), as well as the strongest DPPH scavenging activity (44.51%), while light flour showed the lowest values. The choice of milling method also critically affects phenolic retention in Tartary buckwheat flour. Ultrafine milling (UM) and stone milling (SM) retain higher rutin and TPC compared with roller milling (RM) and wet milling (WM) (F. Liu, He, et al., 2018). UM reduces particle size, increasing compound accessibility, whereas WM favors quercetin over rutin, indicating a shift in flavonoid composition during flour processing (F. Liu et al., 2018). Superfine grinding of Tartary buckwheat bran, combined with endogenous rutin-degrading enzyme catalysis, significantly increases aglycone formation and ethyl-rutinoside levels, enhancing antioxidant and α-glucosidase inhibitory activities (Xiao et al., 2022). Similarly, Q. Xu et al. (2021) further demonstrated that wet-ground bran lost rutin but accumulated quercetin, reaching 2.18 g/100 g, while exhibiting strong radical scavenging activity (DPPH 60.74%, •OH 86.62%, ABTS 92.98%). Particle size also influences the nutritional properties of buckwheat flours. Bani et al. (2025) showed that fine flours retained higher phenolic contents than coarse flours and exhibited stronger antioxidant and antidiabetic activities. Fine flours contained more protein (9.9–13.3 g/100 g) and riboflavin (117–172 μg/100 g), with their phenolic enrichment linked to greater α-glucosidase and α-amylase inhibition. Collectively, these findings demonstrate that milling governs the nutraceutical profile of Tartary buckwheat by determining whether phenolics are preserved, degraded, or transformed. Outer fractions (bran and husk) act as concentrated reservoirs of bioactives, with rutin contents ranging from 360 mg/100 g in flour to over 8600 mg/100 g in bran. Milling method and particle size are decisive factors, with stone and ultrafine milling as well as bran backfilling strategies offering promising approaches to maximize the health-promoting potential of Tartary buckwheat in food applications.

#### 3.1.2 Soaking

**Fig. 1:**
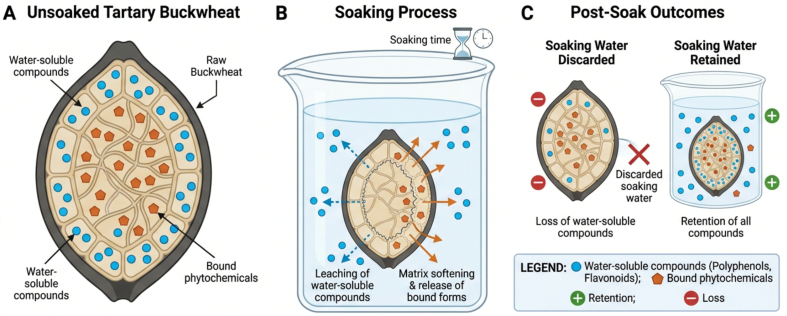
Mechanisms determining retention of polyphenols and flavonoids during soaking in Tartary buckwheat.

Soaking operates primarily as an aqueous pre-processing step in cereals and pseudocereals such as Tartary buckwheat, with dual impacts on polyphenolic content. The impact of soaking on the retention of polyphenols, phenolic compounds, and flavonoids in Tartary buckwheat is determined by two principal mechanisms: (1) the leaching of water-soluble compounds into the soaking medium (Lima et al., 2017); and (2) the enhanced extractability and release of bound forms due to matrix softening. While soaking can facilitate the release of bound phytochemicals, it concurrently exposes water-soluble constituents to leaching, particularly if the soaking water is discarded (Fig. 1). The balance between these two mechanisms is highly sensitive to soaking time, temperature, and whether subsequent processing utilizes or discards the soaking medium. To optimize retention of bioactive compounds in Tartary buckwheat grains, soaking conditions are critical. Brajčič et al. (2020) reported that soaking Tartary buckwheat grains at lower temperatures (21 °C) for short durations (20 min) preserved rutin because rutin-degrading enzymes remained inactive. Similarly, soaking Tartary buckwheat grains at 26 °C for 20 h resulted in a flavonoid content of 13.95 mg g^−1^, indicating that temperature and soaking duration can enhance flavonoid concentration (Y. Guo et al., 2013). However, when soaking temperature increased to 51 °C or higher, grain structures degraded sufficiently to allow rutin-degrading enzymes to interact with their substrate, leading to a decrease in rutin and an increase in quercetin. Heating above 80 °C for 20 min inactivated these enzymes and helped preserve rutin (Brajčič et al., 2020). Moreover, the antioxidant capacity is closely linked to the TPC, which is positively correlated with antioxidant and α-glucosidase inhibition activities (Qin et al., 2013). Kalinová et al. (2019) separately evaluated soaking effects on buckwheat hulls and groats. Soaking hulls for one hour increased rutin content (128.72–174.43 mg kg^−1^ DW), while catechin, epicatechin, hyperoside, and total phenolics decreased by approximately 37%. In contrast, soaking groats for the same duration increased catechin and rutin levels (from 20.87 to 34.89 mg kg^−1^ DW and 52.48 to 55.78 mg kg^−1^ DW, respectively), while epicatechin and epicatechin gallate declined (from 56.51 to 36.85 mg kg^−1^ DW and 9.80 to 9.33 mg kg^−1^ DW, respectively). Extended soaking durations have also been shown to enhance TPC and antioxidant activity in buckwheat grains. Thakur et al. (2021) reported that buckwheat grains soaked for 12 and 24 h had higher TPC (251.87 and 280.81 mg GAE/100 g, respectively) and antioxidant capacity (52.53% and 55.46%, respectively) compared to unsoaked grains (210.31 mg GAE/100 g, 31.69%). Similarly, Brožková et al. (2018) found that soaking common buckwheat groats at 5 °C for 12–24 h increased TPC and quercetin levels while reducing microbial counts. In contrast, Terpinc et al. (2016) reported no significant change in TPC after soaking buckwheat grains for 8 h. Overall, optimizing soaking time and temperature is essential to balance enhanced extractability and prevention of leaching losses, with moderate soaking conditions generally preserving higher levels of bioactive compounds.

#### 3.1.3 Germination

Germination significantly influences the flavonoid content in buckwheat, particularly in Tartary buckwheat. Germination of buckwheat seeds, leading to sprout formation, activates metabolic pathways that enhance the synthesis and accumulation of flavonoids known for their antioxidant properties and health benefits (Dong et al., 2023). One of the key biochemical changes during germination of buckwheat seeds and sprouts is the activation of phenylalanine ammonia-lyase (PAL), an enzyme crucial for the flavonoid biosynthesis pathway. G. Zhang et al. (2015) reported that PAL activity increases during germination, leading to enhanced accumulation of flavonoids such as rutin and vitexin in germinated buckwheat tissues. This increase is attributed to activation of glycosyltransferases, facilitating formation of flavonoid glycosides and enriching the flavonoid profile of germinated buckwheat. During germination of buckwheat seeds into sprouts, marked increases in TPC and antioxidant activity occur, attributed to elevated levels of flavonoids such as rutin, quercetin, vitexin, and isoorientin in sprout tissues (Živković et al., 2021). For instance, rutin content in buckwheat sprouts can reach up to 283.43 mg/100 g dry weight, compared with lower levels in ungerminated seeds (Caterina & Camelia, 2012). This increase in flavonoid content is linked to the activation of the PAL gene, which plays a crucial role in the biosynthesis of phenolic compounds (Ling et al., 2018). Enhanced antioxidant activity is also observed in extracts from germinated sprouts, with significant increases in DPPH and ABTS radical scavenging activities (X. Zhou et al., 2015). Furthermore, the germination of buckwheat seeds decreases anti-nutritional factors, thereby improving the overall nutritional profile of buckwheat (Borgonovi et al., 2023). The increase in flavonoid content and antioxidant activity during germination makes buckwheat sprouts a promising functional food ingredient, offering potential health benefits such as reducing the risk of chronic diseases (Caterina et al., 2012). Additionally, the germination process can be further optimized through techniques like elicitation, which can enhance the production of low-molecular antioxidants and improve the quality of buckwheat sprouts. Germination time is a critical factor influencing flavonoid accumulation. Wiczkowski et al. (2014) reported that the rutin content in buckwheat sprouts increased significantly from 23.4 mg per 100 g of fresh weight at day 3 to a maximum of 109.0 mg per 100 g at day 6. This rapid increase underscores the importance of timing in harvesting buckwheat sprouts to maximize flavonoid content. Similarly, Koyama et al. (2013) found that while certain C-glycosylflavones decreased as the sprouts matured, rutin levels continued to rise, indicating a dynamic change in flavonoid composition during the growth stages. Delayed germination in buckwheat markedly influences its flavonoid content and antioxidant activity, as demonstrated by multiple studies. During a 12-day germination period, rutin levels in common buckwheat cotyledons increased dramatically, reaching 7.7 times those of ungerminated seeds (Ling et al., 2018). Likewise, X. Zhou et al. (2015) reported that after 7 days of germination of buckwheat seeds, TFC and rutin concentration rose to 19.53 mg rutin/g and 11.34 mg g^−1^, respectively, highlighting a substantial accumulation of these bioactive compounds. This enhancement is largely attributed to the activation of phenolic biosynthesis enzymes such as PAL (Y. Chen et al., 2022). Although flavonoid content in Tartary buckwheat sprouts continues to rise up to 10 days of germination, the most pronounced increase generally occurs by day 7, making this stage optimal for maximizing flavonoid yield (J.-b. Wang, Zhang, Peng, Zou, Xiang, & Zhao, 2013). Consequently, a germination period of 7 to 10 days is often recommended, with potential for further enhancement through additional treatments. Notably, the peak accumulation times for flavonoids and rutin can differ. For example, Jia-qian et al. (2023) observed maximum levels on day 6 of germination, with flavonoids at 26.54 mg g^−1^ and rutin at 13.88 mg g^−1^, both significantly exceeding values in ungerminated seeds. Furthermore, combining germination with hydrothermal treatment can accelerate and intensify accumulation, with rutin content reaching 22.31 mg g^−1^ on the first day under this combined treatment, representing a 4.75-fold increase compared with non-germinated samples. This combination also enhances the antioxidant capacity and α-amylase inhibition rate, indicating improved functional properties. The use of innovative physical technologies, such as microwave and ultrasonic treatments, during germination can further promote the enrichment of active substances, including flavonoids, in Tartary buckwheat sprouts (Dong et al., 2023). Microwave and *L*-phenylalanine (L-Phe) treatments can promote flavonoid accumulation in Tartary buckwheat sprouts. Under optimal conditions (microwave 250 W, 90 s, 2.9 mM L-Phe), the specific activities of key enzymes (PAL, CHI, FLS) in 5-day-old sprouts increased by 47.84%, 53.04%, and 28.02% respectively, compared to controls (W. Peng et al., 2023). This co-treatment also increased the expression of related genes (FtPAL, FtCHI, and FtFlS1) by 39.84%, 24.78%, and 33.72% respectively (W. Peng et al., 2023). Post-germination processing methods can further influence flavonoid levels. Different drying approaches, such as hot air drying combined with pretreatments like color protection (CP), osmosis (OM), blanching (BC), β-cyclodextrin (β-CD), and ultrasound (US), have demonstrated varying effects. At 60 °C, TFC in sprouts increased by 8.76% with CP, 6.76% with OM, 12.34% with β-CD, and 4.25% with US, relative to untreated controls (X. Xu et al., 2024). These findings suggest that a combination of controlled germination conditions, hydrothermal treatment, and innovative technologies can effectively maximize the bioavailability of flavonoids and rutin in Tartary buckwheat products.

#### 3.1.4 Fermentation

Fermentation has been applied to Tartary buckwheat as a bioprocessing strategy to modify its bioactive profile, with the most consistently reported changes involving phenolics and flavonoids.

##### 3.1.4.1 Bacterial fermentation

**Fig. 2:**
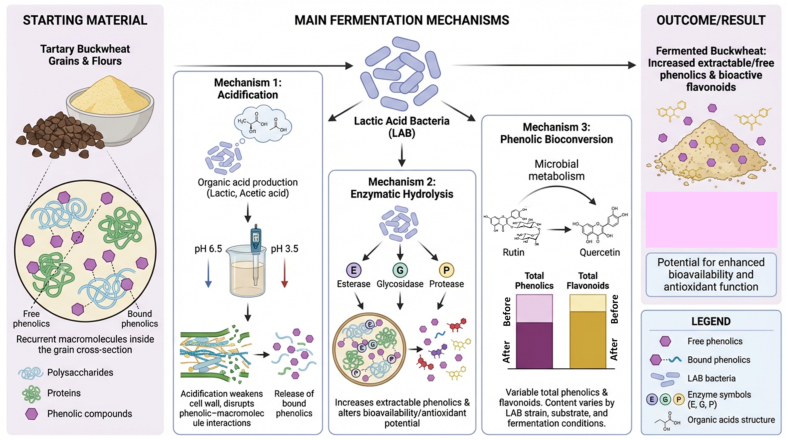
Main mechanisms by which lactic acid bacteria fermentation modifies bioactive compounds in Tartary buckwheat.

Bacterial fermentation, mainly carried out by lactic acid bacteria (LAB), alters the biochemical composition of Tartary buckwheat grains and flours and consequently modifies their bioactive compounds. During fermentation, bacterial metabolism transforms components within the cereal matrix, leading to measurable changes in phenolic composition and associated antioxidant properties (Fig. 2). Solid-state fermentation of Tartary buckwheat flour with *Lactobacillus plantarum* TK9 resulted in an increase in TPC, rising from 243.0 mg gallic acid equivalents (GAE)/g dry extract in unfermented material to 251.8 mg GAE/g after fermentation. In contrast, fermentation with *Lactobacillus paracasei* TK1501 maintained phenolic levels similar to those of native samples, indicating that the impact of bacterial fermentation depends strongly on the LAB strain used. These results demonstrate that LAB activity can either enhance or preserve phenolic compounds through microbial metabolism during fermentation (Feng et al., 2018). The observed increase in phenolic content is attributed to bacterial enzymatic activity, which modifies the levels and forms of bioactive compounds during fermentation. LAB fermentation facilitates the transformation of phenolic compounds present in bound forms within the grain structure into more extractable forms, thereby improving their measurable concentration and antioxidant potential (Feng et al., 2018). Liquid-state fermentation of buckwheat flours using selected LAB strains has also demonstrated modifications in phenolic composition and antioxidant capacity. LAB fermentation generally caused slight, strain-dependent increases in TPC, although rutin content tended to decrease during fermentation. Despite these variations, LAB fermentation overall improved antioxidant and functional properties of buckwheat flours, showing that bacterial fermentation can enhance bioactive potential depending on processing conditions and microbial strains applied (Zieliński et al., 2019). Further evidence shows that bacterial fermentation significantly enhances flavonoid compounds and antioxidant activity, especially when germinated buckwheat grains or sprouts are used as substrates. Fermentation with *Bifidobacterium breve*, *Lactobacillus buchneri*, *Bifidobacterium animalis*, and *Bacillus subtilis* increased rutin content up to 0.97 g kg^−1^ and quercetin up to 2.53 g kg^−1^. Total phenolic compounds increased from 0.2% in raw material to as high as 3.41%, while total flavonoids reached 4.28%. Correspondingly, DPPH and ABTS radical scavenging activities rose to 45.56% and 65.21%, respectively, demonstrating strong enhancement of antioxidant properties after fermentation of germinated buckwheat grains (Ratha & Jhon, 2017). In addition to phenolic modification, LAB fermentation promotes protein transformation in Tartary buckwheat. Fermentation with *Lactiplantibacillus plantarum* significantly increased peptide production by converting native proteins into smaller polypeptides that can be more readily absorbed in the intestine. This transformation improves protein utilization and increases the functional value of fermented buckwheat products (Panpan Wang & Ma, 2024). Overall, bacterial fermentation enhances phenolic extractability, antioxidant activity, enzyme inhibitory potential, and protein digestibility, with outcomes strongly dependent on bacterial strains and fermentation conditions.

##### 3.1.4.2 Fungal fermentation

Fungal fermentation has been widely applied to Tartary buckwheat to enhance bioactive compounds through enzymatic degradation of structural components and metabolic transformation of phytochemicals, resulting in improved antioxidant and physiological properties. Solid-state fermentation using *Eurotium cristatum* YL-1 produced pronounced metabolite changes compared with non-fermented samples, with numerous metabolites differing after fermentation. Most phenolic compounds increased, accompanied by significant enhancement of antioxidant capacity and α-glucosidase inhibitory activity, demonstrating improved anti-glycemic potential after fermentation (Xiao, Wu, et al., 2021). Similarly, fermentation with *Monascus purpureus* significantly increased bioactive constituents in whole-grain Tartary buckwheat. Total phenols and flavonoids increased by 24.97% and 45.74%, respectively, and rutin content reached 2607.31 mg per 100 g dry weight. Correspondingly, antioxidant activities measured by DPPH, ABTS, and FRAP assays were significantly enhanced, and fermented products showed improved physiological responses including modulation of lipid metabolism and intestinal microbiota, indicating increased functional value of fermented buckwheat products (Yang et al., 2024). Additional evidence from fermentation with filamentous fungi further confirms the enhancement of phenolic compounds and biological activity. Solid-state fermentation using *Rhizopus oryzae* 40,469, *Rhizopus oryzae* 40,503, and *Rhizopus oligosporus* 3152 significantly increased phenolic and flavonoid contents compared with native Tartary buckwheat. TPC increased from 36.80 μmol g^−1^ in unfermented samples to 93.11, 85.17, and 68.66 μmol g^−1^ after fermentation with the respective strains, while total flavonoid content increased from 13.64 to 31.04, 24.87, and 19.08 μmol g^−1^. Rutin content approximately doubled following fermentation (Huang et al., 2017). Fermented extracts also provided stronger protection against AAPH-induced DNA strand damage and significantly inhibited nitric oxide production and iNOS gene expression in macrophage cells, demonstrating enhanced antioxidant and anti-inflammatory properties after fermentation (Huang et al., 2017). Fermentation with *Ganoderma lucidum* further demonstrated strong modification effects on Tartary buckwheat composition. Activities of carbohydrate-hydrolyzing enzymes increased markedly during fermentation, resulting in increases of polysaccharides, total phenols, flavonoids, and triterpenoids by 122.19%, 113.70%, 203.74%, and 123.27%, respectively, accompanied by significantly improved antioxidant and anti-glycemic activities (R. Zhang et al., 2024). In summary, fungal fermentation significantly enhances phenolic availability, antioxidant capacity, enzyme inhibitory activity, and functional polysaccharide properties of Tartary buckwheat through enzymatic degradation and metabolic conversion, demonstrating its effectiveness in producing value-added buckwheat foods with improved bioactive and physiological properties.

### 3.2 Thermal processing

#### 3.2.1 Boiling

Boiling has a significant impact on the retention of flavonoids in Tartary buckwheat, particularly rutin, which is a major flavonoid in this grain. Studies conducted on Tartary buckwheat grains and grain-derived products indicate that boiling is among the thermal treatments that best preserve flavonoids, particularly rutin (Jin et al., 2020; Y. Liu et al., 2019). In experiments using Tartary buckwheat seeds, boiling and steaming caused smaller decreases in total phenolic content (TPC) and total flavonoid content (TFC) compared with roasting or microwave treatments, which resulted in larger losses (Y. Liu et al., 2019). Short-duration boiling applied to Tartary buckwheat seeds, for example 90 s, retained more than 85% of the initial rutin content. This preservation effect is attributed to rapid thermal inactivation of rutin-degrading enzymes, which limits the conversion of rutin to quercetin and reduces bitterness (D. Li et al., 2008). The stability of rutin during boiling is attributed to the glycosidic bonds in its structure, which enhance its resistance to degradation in water (Lin et al., 2023). Additionally, hydrothermal treatments, including boiling, have been shown to enhance the antioxidant activity and intestinal absorption of rutin, suggesting that such treatments not only preserve but may also enhance the bioavailability of flavonoids in Tartary buckwheat (Jin et al., 2020). Therefore, boiling is recommended as a favorable method for processing Tartary buckwheat to maximize the retention of its beneficial flavonoids, particularly rutin, while maintaining its nutritional and functional properties. The stability of Tartary buckwheat flavonoids is significantly influenced by different boiling times and temperatures, as evidenced by various studies. For instance, hydrothermal treatment at 100 °C for 20 min was found to be optimal for preserving rutin in Tartary buckwheat enriched dough and Chinese steamed bread, compared to other durations and higher temperatures, which led to greater losses of flavonoids (X. Wang et al., 2017). Furthermore, boiling at temperatures above 80 °C for 20 min was shown to deactivate rutin-degrading enzymes, thereby preserving rutin content in Tartary buckwheat grains (Brajčič et al., 2020). This is crucial as the enzymatic degradation of rutin to quercetin is a key factor in flavonoid stability. Additionally, recent work on Tartary buckwheat sprouts shows that boiling at approximately 100 °C causes progressive decreases in total flavonoids and phenolics as cooking time increases, mainly due to leaching of water-soluble compounds and thermal degradation. For example, flavonoid content decreased notably after 30–90 s of boiling, accompanied by reduced antioxidant activity, although short boiling times may improve extractability of pigments such as chlorophyll (N. Wang, Dong, et al., 2024). The conflicting findings highlight the need for a nuanced understanding of how boiling affects flavonoids in Tartary buckwheat. While the general consensus points to a decrease in TFC due to thermal degradation and leaching, the potential for increased bioavailability of certain flavonoids complicates the narrative. This duality suggests that the cooking method and conditions (e.g., temperature and duration) play critical roles in determining the final flavonoid profile and antioxidant capacity of boiled Tartary buckwheat.

#### 3.2.2 Steaming

Steaming, as a hydrothermal treatment, is a critical processing method for Tartary buckwheat because it modulates flavonoid stability, phenolic retention, and antioxidant activity. Compared with harsher dry-heat methods such as roasting, steaming generally offers a moderate processing environment that both inactivates deleterious enzymes and preserves bioactive compounds. The outcomes, however, are dependent on processing parameters such as time, temperature, and whether steaming is applied alone or in combination with other treatments. Steaming markedly affects the TPC and TFC of Tartary buckwheat. Y. Liu et al. (2019) reported that steaming, along with boiling, promoted increases in TPC, TFC, and antioxidant indices (DPPH, FRAP, ABTS) in Tartary buckwheat seeds, in contrast to roasting or microwaving, which caused significant degradation. Similarly, Xiaojiang Wu et al. (2020) observed that saturated steaming (ST) effectively inactivated rutin-degrading enzymes (RDEs), leading to a 12.3% increase in extractable rutin, while superheated steam achieved even higher improvements in rutin and soluble phenolic content. In buckwheat bran, steam explosion pretreatment further enhanced the release of bound phenolics such as caffeic and protocatechuic acids, resulting in increased antioxidant capacity (W. Li et al., 2020). By contrast, steaming may also induce partial degradation when prolonged. S. Chen et al. (2021) found that steaming significantly decreased phenolic compounds and antioxidant activity in both common and Tartary buckwheat varieties. Qin et al. (2013) provided mechanistic insight, showing that rutin decreased during soaking but increased again after steaming due to a reverse catalytic reaction favoring rutin synthesis. These findings suggest that steaming duration is a key determinant in balancing preservation versus degradation of phenolics. Rutin, the dominant flavonoid in Tartary buckwheat, is highly susceptible to hydrolysis into quercetin by endogenous rutinosidase, generating bitterness and decreasing bioavailability (Suzuki et al., 2021). Hydrothermal processes such as steaming effectively inactivate this enzyme, thereby stabilizing rutin content. Lukšič et al. (2016) demonstrated that hydrothermal treatment embedded rutin within the starch–protein matrix, slowing its release but protecting it from enzymatic conversion to quercetin during bread making. Tang et al. (2024) demonstrated that steaming followed by oven-drying (S + OD) of buckwheat flour led to the highest rutin retention and the lowest quercetin accumulation compared with roasting or stir-frying treatments. Likewise, X. Wang et al. (2017) identified that hydrothermal pretreatment at 100 °C for 20 min provided the optimal balance between rutin preservation, reduced bitterness, and acceptable dough properties for Chinese steamed bread. Xiao, Yang, et al. (2021) further demonstrated that during steamed bread preparation, antioxidant capacity and α-glucosidase inhibitory activity were elevated after dough formation and fermentation, and although they slightly decreased after steaming, they remained higher than in raw flour. This indicates that steaming not only preserves but also enhances bioactivity under controlled conditions. The mechanisms underlying these outcomes are attributed to structural modifications in the grain matrix and enzyme inactivation. Steaming alters starch crystallinity, gelatinization, and retrogradation, which in turn modulate the release and stability of phenolics. For instance, autoclaving and steaming were shown to increase starch digestibility while simultaneously stabilizing flavonoid–starch complexes, thereby influencing both nutritional and functional properties (F. Zheng et al., 2023). Moreover, steaming reduces rutin hydrolysis and bitterness, which improves sensory acceptance of steamed bread and noodles (Tang et al., 2024; X. Wang et al., 2017). When compared with roasting, microwave cooking, and infrared drying, steaming consistently emerges as the more favorable method for retaining bioactive compounds. M. Zhang et al. (2010) reported that pressured-steam heating caused less decreases in phenolics and antioxidant activities than roasting or microwave heating, emphasizing its relative gentleness. In summary, steaming exerts complex but generally positive effects on the bioactive compounds and antioxidant capacity of Tartary buckwheat. Optimized steaming parameters inactivate rutin-degrading enzymes, limit the rutin-to-quercetin transformation, and enhance phenolic release, thereby preserving antioxidant activity. Excessive steaming time, however, can induce losses in phenolics. Compared with other thermal processes, steaming offers the best compromise between functional preservation and sensory quality, making it a recommended approach for developing high-value Tartary buckwheat-based foods.

#### 3.2.3 Roasting

Roasting is a widely applied processing method that significantly alters the functional components of Tartary buckwheat (*Fagopyrum tataricum*). The degree of these changes is determined by key parameters such as roasting temperature, duration, and heating technique, all of which influence the levels of TPC, TFC, and the antioxidant potential of the grain (Klepacka & Najda, 2020). Studies employing infrared roasting have shown that moderate heating conditions, typically in the range of 130–150 °C, are relatively effective in preserving flavonoids in whole Tartary buckwheat seeds, whereas higher roasting temperatures, around 170 °C, lead to pronounced losses of phenolic compounds (Bhinder et al., 2019). Both total phenolic content and overall antioxidant activity were reported to decrease progressively with increasing roasting temperature. Among individual polyphenols, rutin exhibited the highest thermal stability and remained detectable across different roasting conditions, while other phenolics, including gallic acid and quercetin, were more susceptible to thermal degradation (Bhinder et al., 2019). Industrial-scale investigations using whole Tartary buckwheat grains further confirmed that roasting reduces phenolic compounds, particularly rutin and *p*-coumaric acid, with bioactive losses driven primarily by thermal intensity rather than roasting duration. These findings suggest that maintaining lower roasting temperatures (close to 100 °C) for longer times may better preserve phenolics while still improving sensory attributes (Klepacka et al., 2020). When roasting was applied to germinated Tartary buckwheat grains, time-dependent effects were observed. Koo et al. (2023) reported that roasting germinated grains at 200 °C for short durations (approximately 7 min) transiently increased phenolic and flavonoid contents, whereas prolonged roasting (15–21 min) caused marked decreases in rutin, total flavonoids, γ-aminobutyric acid (GABA), and antioxidant activity. Antioxidant capacity followed a similar trend, with a brief enhancement followed by substantial decrease under extended heat exposure. Comparative studies using whole Tartary buckwheat seeds indicate that roasting generally causes greater degradation of phenolics and flavonoids than hydrothermal treatments such as boiling or steaming, which more effectively preserve bioactive compounds (Y. Liu et al., 2019). This highlights the stronger destabilizing effect of dry heat relative to moist heat on seed-bound phenolics. In contrast, investigations focusing on Tartary buckwheat husks revealed that roasting can increase the extractability of bound phenolics. A. Liu et al. (2025) showed that roasting husk material enhanced measurable phenolic content and antioxidant activity, likely due to disruption of the lignocellulosic matrix and release of bound compounds. Cross-species evaluations also demonstrate differences in response to roasting. When exposed to short-term high-temperature roasting (200 °C for 50 s), Tartary buckwheat grains showed significant decreases in phenolics, flavonoids, and antioxidant activity. In contrast, some common buckwheat varieties exhibited slight increases in TPC under the same conditions, reflecting their comparatively greater thermal stability (Ma et al., 2020). Finally, studies examining roasted Tartary buckwheat seeds processed into infusion teas demonstrated that post-roasting extraction conditions strongly influence bioactivity. Ryu, Choi et al. (2020) reported that roasted Tartary buckwheat seeds brewed under cold-extraction conditions yielded higher polyphenol and flavonoid levels and stronger antioxidant and antiproliferative activities than hot-brewed counterparts. In summary, roasting applied to whole seeds, germinated grains, or husk fractions of Tartary buckwheat generally reduces phenolic content and antioxidant capacity as roasting intensity increases. Moderate roasting can transiently enhance extractability or preserve rutin, but excessive temperature and duration promote degradation. While roasting improves sensory quality and reduces bitterness, careful optimization of roasting parameters and clear identification of the treated material are essential to balance nutritional retention with product acceptability.

#### 3.2.4 Extrusion

Extrusion has been widely applied to Tartary buckwheat flour and flour-based formulations, where thermal and mechanical stresses modify phytochemical stability, transformation, and antioxidant capacity. Most extrusion studies on Tartary buckwheat have been conducted using whole meal flour or composite flours, rather than intact grains. In Tartary buckwheat, extrusion parameters such as temperature, feed moisture, screw speed, and post-processing treatments directly influence the retention, transformation, and bioavailability of bioactive compounds, thereby modulating the antioxidant potential of the final products. Using pregelatinized Tartary buckwheat flour, Z. Zhang et al. (2022) showed that increasing extrusion temperature led to decreases in total flavonoids and rutin, while simultaneously promoting the conversion of flavonoid glycosides into aglycones such as quercetin and kaempferol. These transformations enhanced antioxidant and α-glucosidase inhibitory activities, indicating that although total flavonoid content declined, bioactivity increased due to aglycone formation. Similar trends were reported by Beitane, Krumina Zemture & Sabovics (2018) using Tartary buckwheat flour, where extrusion at elevated temperatures reduced total phenolic content and antioxidant activity, but also generated new phenolic acids, including ferulic acid and hyperoside, reflecting structural rearrangement of native compounds. Feed moisture during extrusion plays a protective role, particularly in flour-based systems. Using Tartary buckwheat flour, Cheng et al. (2020) demonstrated that improved extrusion cooking technology with higher feed moisture retained more phenolics than conventional low-moisture extrusion. Likewise, Q. Wang et al. (2020) reported that noodles prepared from extruded Tartary buckwheat–wheat composite flour showed higher TPC, TFC, rutin, and antioxidant activity when processed at higher moisture levels, due to reduced thermal and shear stress. Shear intensity further influences phytochemical stability in buckwheat flour matrices. Z. Zhang et al. (2022) showed that traditional high-shear extrusion caused extensive degradation of phenolics in Tartary buckwheat flour, whereas lower-shear configurations preserved more bioactive compounds. These findings indicate that screw design and rotational speed are critical variables when extruding buckwheat flour. Biological fortification during extrusion has also been explored in buckwheat flour systems. Park et al. (2022) demonstrated that hot-melt extrusion of buckwheat flour supplemented with yeast significantly increased TPC, TFC, rutin, and antioxidant activity compared with non-extruded flour, highlighting synergistic effects between extrusion and microbial components. Extrusion effects also depend on formulation context. Fu et al. (2020) observed that partial substitution of rice flour with buckwheat flour (30 g/100 g) in extruded noodles significantly increased polyphenol and flavonoid contents compared to pure rice noodles, with higher retention of these compounds after extrusion cooking. Singh et al. (2019) confirmed that extrusion temperature modulates these outcomes, showing that increasing extrusion temperature enhanced antioxidant activity but reduced phenolic content in corn–buckwheat extrudates. These findings underscore the complex interplay between processing parameters, formulation, and health-relevant outcomes. While extrusion can enhance aglycone formation and antioxidant capacity, negative sensory implications must also be considered. Overall, extrusion studies on Tartary buckwheat have been conducted predominantly on wholemeal flour or composite flour systems, rather than intact grains. High temperature and shear generally reduce total phenolics and flavonoids, while promoting aglycone formation and bioactivity. Feed moisture, screw configuration, and formulation composition critically determine whether extrusion acts primarily as a degradative or functional transformation process. Careful optimization of extrusion conditions is therefore essential to balance phytochemical retention, bioactivity enhancement, and sensory quality in Tartary buckwheat flour–based products.

## 4 Challenges and future work

The effects of processing on flavonoid concentration and transformation in Tartary buckwheat are complex and often inconsistent. Techniques including milling, soaking, germination, fermentation, and thermal treatments (boiling, steaming, roasting, extrusion) can either enhance bioavailability or promote degradation, depending on conditions. Milling increases flavonoid accessibility by disrupting the seed coat but can also cause losses through mechanical damage. Soaking facilitates the migration of rutin from bran to endosperm, enriching the flour, yet it may activate enzymes that convert rutin to quercetin; to stabilize the profile, soaking is often followed by heat treatments that inactivate these enzymes. Germination can activate metabolic pathways that improve antioxidant properties, although its effect on flavonoid profiles varies with buckwheat variety and germination conditions. Fermentation may increase the concentration and solubility of flavonoids, but outcomes depend on microbial strains, fermentation time, and environmental factors. Thermal treatments improve sensory characteristics but can reduce flavonoid levels, especially at high temperatures. While some studies report substantial rutin loss during heating, others find minimal change; these discrepancies likely reflect differences in temperature, duration, and analytical methods. The absence of standardised processing and analytical protocols contributes to variability in reported results. Some processes also convert flavonoids into derivatives such as quercetin, which may have distinct bioactivities, yet the overall impacts on bioavailability and health remain insufficiently understood. This is further complicated by individual health conditions (for example diabetes or hypertension), which may influence physiological responses to processed buckwheat products. To address these challenges, future research should prioritise standardised conditions for each processing technique, defining specific parameters (temperature, time, moisture) to ensure consistent flavonoid retention and functional quality. Combinations of processes, such as milling followed by fermentation or germination followed by steaming, should be explored for potential synergistic gains in nutritional value and sensory quality. Gentle heating strategies (for example low-temperature roasting, microwave heating, infrared processing) may preserve more flavonoids than conventional high-temperature methods. Fermentation should be optimized through careful selection of microbial strains and control of key parameters to promote conversion into more bioavailable forms, while also assessing effects on other health-related compounds such as phenolic acids and saponins. A more complete biochemical understanding is essential: future work should map molecular transformations of flavonoids during thermal and fermentation treatments, identify the specific derivatives formed, evaluate their health effects, and clarify the enzymatic pathways involved. These insights will guide the development of high-quality Tartary buckwheat products with improved nutritional and functional properties.

## 5 Conclusions and outlook

Tartary buckwheat is a rich source of flavonoids, particularly rutin, with notable variations in content across different plant parts and varieties. The stability and bioactivity of these flavonoids are influenced by factors such as genetic traits, environmental conditions, and processing techniques. Understanding these factors is crucial for optimizing the health benefits of Tartary buckwheat in functional food applications. Processing methods such as milling, soaking, germination, fermentation, boiling, steaming, roasting, and extrusion play key roles in shaping the flavonoid profile. Milling and germination enhance flavonoid retention and bioavailability, while fermentation can alter the qualitative properties and antioxidant activities, improving the functional potential of Tartary buckwheat. Soaking improves flavonoid availability in Tartary buckwheat but may trigger rutin degradation. Follow-up thermal treatments help stabilize rutin, enhancing flavonoid retention in the final product. Thermal treatments like boiling and steaming, when properly optimized, help preserve flavonoids, particularly rutin, and enhance antioxidant properties. Roasting and extrusion also offer benefits, though they may involve trade-offs in flavonoid retention versus antioxidant activity. The impact of these processes on flavonoid content underscores the need for careful optimization to maximize health benefits. Furthermore, the storage conditions of Tartary buckwheat are essential for maintaining flavonoid stability, with high temperatures, humidity, and light exposure potentially leading to degradation. Future research should focus on refining processing parameters and storage strategies to enhance flavonoid retention, bioavailability, and overall nutritional value. By improving these factors, Tartary buckwheat has the potential to be a highly functional and health-promoting food source.

## CRediT authorship contribution statement

**Hong-Ju He:** Writing – review & editing, Resources, Formal analysis, Conceptualization. **Guanglei Li:** Writing – review & editing, Supervision, Data curation. **Xingqi Ou:** Writing – review & editing, Project administration, Funding acquisition. **Amer Ali Mahdi:** Writing – review & editing, Software, Resources. **Mohammed Obadi:** Writing – original draft, Investigation, Conceptualization.

## Declaration of competing interest

The authors declare that they have no known competing financial interests or personal relationships that could have appeared to influence the work reported in this paper.

## Data Availability

No data was used for the research described in the article.
